# Medical Liability of Residents in Taiwan Criminal Court: An Analysis of Closed Malpractice Cases

**DOI:** 10.1155/2020/7692964

**Published:** 2020-06-01

**Authors:** Kuan-Han Wu, Po-Chun Chuang, Chih-Min Su, Fu-Jen Cheng, Chien-Hung Wu, Fu-Cheng Chen, Yii-Ting Huang

**Affiliations:** Department of Emergency Medicine, Kaohsiung Chang Gung Memorial Hospital, Chang Gung University College of Medicine, No. 123, Dapi Rd., Niaosong Dist., Kaohsiung City 833, Taiwan

## Abstract

**Objective:**

By analyzing closed criminal malpractice claims involving resident physicians, we aimed to clarify the characteristics of litigations and examine the litigious errors leading to guilty verdicts.

**Design:**

A retrospective descriptive study. *Setting/Study Participants*. The verdicts pertaining to physicians recorded on the national database of the Taiwan justice system were reviewed. *Main Outcome Measures*. The characteristics of litigations were documented. Negligence and guilty verdicts were further analyzed to identify litigious errors.

**Results:**

Between January 1, 2000, and December 31, 2014, from a total of 436 closed criminal malpractice cases, 40 included resident physicians. Five (12.5%) cases received guilty verdicts with mean imprisonment sentences of 5.4 ± 4.1 months. An average of 77.2 months was required for the final adjudication, and surgery residents were involved most frequently (38.9%). Attending physicians were codefendants in 82.5% of cases and were declared guilty in 60% of them. Sepsis (37.5%) was the most common disease in the 40 cases examined, followed by operation/procedure complications (25%). Performance errors (70%) were more than twice as common than diagnostic errors (30%), but the percentage of guilty verdicts in performance error cases was much lower (7.1% vs. 25%). Four negligence cases received nonguilty verdicts, which were mostly due to lack of causation.

**Conclusion:**

Closed criminal malpractice cases involving residents took on average 6.22 years to conclude. Performance errors accounted for 70% of cases, with treatment of sepsis and operation/procedure complications predominant. To reduce medicolegal risk, residents should learn experiences from analyzing malpractice cases to avoid similar litigious pitfalls.

## 1. Introduction

The number of medical malpractice cases has increased over the past decades, and resident physicians are frequently brought into lawsuits as codefendants with attending physicians. Residents are involved in 4–26% of medical malpractice lawsuits in previous studies [1–4].

Lawsuits against resident physicians may cause a significant amount of stress and have various consequences. First, litigation may cause direct monetary loss from liability compensation and the risk of being sentenced to imprisonment on a criminal charge. Second, lawsuits require an average of 50.7–58.0 months to conclude [5–7], which means that stressful cases may remain unresolved over the whole residency training program. Third, 95% of physicians report some sort of emotional distress during the litigation process [8]. Eventually, liability-related issues have major effects not only on resident physicians' daily practice but also on the departments and locations where they choose to practice [2, 9]. Moreover, attending physicians and teaching hospitals could be also held liable due to insufficient supervision when malpractice complaints are filed on residents [10, 11].

The risk for physicians to be sued during their career was estimated to be 75% in low-risk specialties and 99% in high-risk specialties [12]; thus, medicolegal issues are almost inevitable. It is also clear that residents cannot escape from judicial culpability in medical malpractice claims simply because of still being in training. Although 96% of residents reported being concerned about malpractice [13], and malpractice reform was one of their top health policy priorities [14], educational programs to prepare them for litigation are lacking [15, 16]. Malpractice and legal training during residency mainly involve morbidity and mortality conferences and lectures on the legal system [17].

Although medicolegal liability in malpractice claims against residents has been described in the previous literature, further analysis of court verdicts pertaining to residents was either limited to a specific specialty in a single institution [1], focused on indemnity payment in common law [4], or studied long time ago [18]. Criminal litigation generates even more stress for resident physicians than monetary compensation because of the risk of imprisonment, but studies have been scarce. Detailed factors of the criminal legal process such as duration have not been identified either.

It is believed that resident physicians may gain knowledge from the analysis of previous criminal malpractice verdicts, which can offer valuable information regarding litigious errors in clinical practice [19, 20]. Therefore, using the national judicial databank of Taiwan, the purpose of this study was to analyze closed criminal malpractice cases pertaining to resident physicians, determine the characteristics of the legal process, and examine the errors that led to guilty verdicts.

### 1.1. Criminal Justice System in Taiwan

The criminal justice system in Taiwan comprises the Supreme Court, High Courts, and District Courts, with a “three-level and three-instance” system. Cases are heard by a District Court first and closed if the Supreme Court affirms the verdict of the High Court or if there is no appeal to a higher court. Verdicts are remanded to the High Court by the Supreme Court if there is doubt regarding jurisdiction and appealed to the Supreme Court if litigants are still against the judgment of the High Court. According to Taiwan's Code of Criminal Procedure, most criminal cases involving healthcare providers are investigated by an official prosecutor. The prosecutor collects detailed evidence and decides whether the case should be sent for indictment. If the court or prosecutor needs testimony to clarify the medical process, the medical records are sent for medical appraisal to decide if the clinical practice deviated from the proper care standards. The court would make final adjudication based on criminal law statute and appraisal opinion.

In Taiwan teaching hospitals, residents and attending physicians collaborate to care for patients, with physicians' ultimate duty being patient care. Residents are responsible for the initial assessment and treatment of patients, and attending physicians need to monitor the whole treatment plan and adjust the decisions of residents if indicated. The residents are obligated to report to and discuss with the attending physician if the clinical situation is uncertain or problems arise. Since the duty of residents and attending physicians is different, prosecutors will investigate the clinical behavior of both and will file charges against them if their practice is deemed inappropriate. The court will return the final verdict after hearing the evidence provided by prosecutor and plaintiffs. Since criminal law in Taiwan regulates the behavior of individuals only, the hospital itself is responsible only for the indemnity rule by civil law and cannot be the defendant in a criminal court.

## 2. Methods

### 2.1. Study Design

A medical malpractice claim was defined as a lawsuit against a healthcare provider that was filed by a patient or patient's family for injury arising from medical care. We conducted a retrospective study reviewing the Taiwanese criminal court verdicts involving residents from 2000 to 2014. Undergraduate medical students were not included because they are not legally certified for clinical practice and every action should be confirmed by supervisors. The study was approved by the institutional review board of our institution.

### 2.2. Study Setting and Database

The Law and Regulations Retrieving System of the Judicial Yuan of the Republic of China is a national deidentified electronic database maintained by Taiwan's justice department. The database includes all criminal litigation verdicts since 2000 after judgment has been passed by District Courts, High Courts, or the Supreme Court. To gather closed malpractice claims involving residents, we searched the database for verdicts of District Courts, High Courts, and the Supreme Court from January 1, 2000, to December 31, 2014, using the keywords “vocational negligence” and “physician,” as “vocational negligence” is the term for malpractice in the Taiwanese criminal justice system. Cases were considered closed if they were ruled by the Supreme Court or there was no further appeal after District Court or High Court adjudication before June 30, 2016. Closed malpractice cases were included in the study if a resident was involved as one of the defendants. The included cases from the District Courts, High Courts, and Supreme Court were then examined to identify the complete appeal process.

### 2.3. Data Collection and Outcomes

The primary outcomes were the characteristics of the legal process and of the primary dispute leading to lawsuit. Collected data included the number of resident and attending physicians involved, specialty of the resident, level of the involved medical institution, diagnosis and outcome of the alleged injury, court-rendered final judgments, results of adjudication, and time elapsed between incident and litigation closure. Four levels of hospital classification (medical centers, regional hospitals, district hospitals, and clinics) were identified based on the Taiwanese accreditation system. The resident physicians were all under training in either regional hospitals or medical centers according to the resident training program designed by specialty societies of different clinical specialties in Taiwan. The final judgments were passed by either the Supreme Court, High Courts, or District Courts depending on whether there was any appeal process.

The specialties of the residents were categorized as follows: (1) internal medicine (cardiovascular, pulmonary, general medicine, infection, nephrology, gastrointestinal, hematology, oncology rheumatology, and metabolic-endocrine); (2) surgery (general surgery, neurosurgery, orthopedic surgery, proctology, plastic surgery, cardiovascular surgery, and anesthesiology); (3) obstetrics and gynecology; (4) pediatrics; (5) emergency medicine; and (6) others. The outcome of injuries was categorized into three levels of severity: (1) death; (2) grave injury, such as brain injury that causes a vegetative state; and (3) other, including all injuries ranging from moderate to mild physical damage or disabilities. In pregnant patients with both maternal and fetal injuries, the more severe one was selected.

Primary dispute was defined as the single most significant argument of plaintiffs that led to litigation. Primary disputes were categorized into two major types: diagnostic error and performance error. Diagnostic errors were those in which the initial diagnosis was not the same as the final diagnosis of the disease that caused the alleged injury. Performance errors were those in which the diagnosis was correct, but the patient was treated in an inappropriate manner in the plaintiff's view with respect to failure to provide adequate treatment, inappropriate medication dosage, inadequate monitoring and supervision, or failure to order consultation. Failure of early recognition of complications of surgery or invasive procedures was classified as a diagnostic error. The categories of primary dispute were determined by two coauthors independently after a review of the verdicts. The final decision on inconsistent cases was made after a consensus meeting with a third reviewer. Three verdicts reviewers were all senior qualified emergency physicians with more than 10 years' clinical experience.

### 2.4. Data Analysis

Descriptive statistics were used to interpret the data. Data were presented as mean ± standard deviation and percentages (%). Comparison of continuous variables between groups was made using Student's *t*-test. A *p* value of less than 0.05 was considered statistically significant.

## 3. Results

All 4937 verdicts extracted from the database between January 1, 2000, and December 31, 2014, were reviewed. A total of 436 closed criminal malpractice cases including 1087 verdicts were identified after tracing appeal processes. From these, 40 cases involving resident physicians (9.2%), including 114 verdicts, were analyzed as final study cases. Attending physicians were involved as codefendants in 33 cases (82.5%). The number and percentage of residents for each specialty group in the 40 malpractice claims are presented in Table 1. There were 21 (38.9%), 13 (24.1%), and seven (13%) residents specializing in surgery, internal medicine, and emergency medicine, respectively.

The basic characteristics of the 40 malpractice claims are presented in Table 2. In total, 35 (87.5%) cases were settled in favor of the clinician. Of the five cases where residents were found guilty, residents' mean imprisonment sentence was 5.4 ± 4.1 months. Despite being found guilty, all residents avoided imprisonment via probation or payment of forfeit. Attending physicians were declared guilty in three cases out of five cases where residents were found guilty (60%). The mean time elapsed between the incident and litigation closure was longer in guilty verdicts than in acquitted cases (95.4 ± 27.9 vs. 74.6 ± 34.5 months), but this difference was not significant (*P*=0.092). Most patients' outcome was death (37 cases, 92.5%), and three cases (7.5%) experienced grave injury. From all verdicts related to patients' death, five cases were claimed as clinicians' lost (13.5%). Twenty-five cases involved medical centers (62.5%), but the percentage of lost claims was higher in regional hospitals.

All cases were sent for medical appraisal, and appropriate treatments were found in 26 cases (65%), with all being acquitted. Seven cases were deemed as negligent behavior by medical appraisal, but four of these were acquitted. A summary of negligent behavior for these acquitted cases is shown in Table 3. Three were acquitted due to lack of causation between negligent behavior and adverse outcome; in the fourth case, the resident's negligent behaviors were under the direct instruction of the attending physician; hence, the attending physician was found guilty.

The number and percentage of disease cases resulting in medical dispute categorized by primary dispute type are presented in Table 4. The rate of performance error was more than twice the rate of diagnostic error (28 cases vs. 12 cases, 70% vs. 30%), but the percentage of guilty verdicts in performance error cases was much lower (7.1% vs. 25%). Overall, sepsis (15 cases, 37.5%) was the most common disease in the examined cases followed by operation/procedure complications; together, they accounted for 20 cases (71.4%) of performance errors. The imprisonment sentences and summary of negligent behaviors for the five cases where residents were found guilty are presented in Table 5.

## 4. Discussion

Although resident physicians are not qualified specialty physicians, they may still face criminal charges if there is negligent practice, while hospital and attending physicians could also be held liable due to responsibility in supervision [17, 21]. In our study, attending physicians were codefendants in 82.5% of cases and were found guilty in 60% of these. In an era of increasing medical malpractice [12], physicians need to recognize the most common characteristics of litigation cases, especially those leading to guilty verdicts. Previous studies suggested that understanding how medical malpractice occurs and is resolved is important [15]. By analyzing closed criminal malpractice cases involving resident physicians over 15 years, especially those leading to guilty verdicts, this study yields several important findings.

First, 70% of malpractice disputes in our study involved performance errors, especially sepsis and operation/procedure complications. However, only 14.3% of these cases had the negligent behavior confirmed by appraisal, and the percentage of guilty verdicts among them was only 7.1%. Why are there malpractice claims in cases without obvious negligence? In our opinion, this is possibly caused by the gap between the expectations of the plaintiff and physician knowledge, as well as lack of communication. For example, postoperative complications might develop in certain cases even though the treatment was flawless. This fact is well known by all physicians, but most families still thought that the complications could have been avoided if the care had been better. In most verdicts, plaintiffs questioned physicians for recklessness in preventing these complications and often accused them regarding various aspects of the medical process, including frequency of staff evaluation, timing of exam, method of surgery, or medication. Plaintiffs are aware that complications might develop, but litigation still occurs if they subjectively consider that a certain action in the treatment was inadequate. By analyzing the verdicts, we believe that the best way to prevent these medical disputes is to communicate, recognize, and pay more attention to what the patient and family really care about. A written informed consent form only might be insufficient to convince plaintiffs that the medical treatment was appropriate.

Second, physicians should be aware that the recent legal standards for resident physicians state that attending physicians need to directly supervise residents' professional conduct [21] although this has been debated [11, 22]. Mostly, resident physicians practice independently without routinely requesting supervision from attending physicians, especially in situations where they are confident and subjectively competent. However, according to our study, if there is negligent conduct, not only the resident physicians but also their supervisor will be held accountable, even the attending physicians never evaluated the patient. The court places the duty on attending physicians to instruct, supervise, and correct residents' clinical performance [11]. Attending physicians will be deemed by the court to incur in a breach of duty if they do not provide appropriate supervision. For ethical purposes and from the patient's perspective, it is also questionable if decisions are made by resident physicians alone, who are not as experienced and qualified as specialty physicians. Some resident physicians tend to handle medical situations by themselves, even critical ones, so as not to “bother” attending physicians, especially when their supervisor is busy. However, this could create potential legal risks for both resident and attending physicians and pose threat to patient safety. Knowledge of these risks should lead resident physicians to proactively seek timely assistance and supervision whenever the clinical situation is difficult or beyond their training level.

Third, legal liability applies only if the four statutory components are owed by plaintiffs, namely, damage, duty of care, breach of duty, and causation [10]. Damage and duty of care in malpractice claims were mostly obvious; thus, the substantial determining factors were whether the physician was negligent (breach of duty) and whether the wrongful act actually caused patient damage (causation). Physicians usually overlook causation, partially because the concept has been emphasized in legal proceedings but not in clinical practice. To clarify, causation is beyond the scope of our manuscript. However, the “But For” test is a simple method for differentiation: “But for the existence of physician's negligence, would patient damage have occurred?” If the answer is no, then the negligence would be the cause of harm, leading to physician's liability. To illustrate this concept, we summarized three cases of negligence by medical appraisal that did not get a guilty verdict because of lack of causation (Table 3). Physicians are not guilty if causation is not established, even if clinical management is definitely negligent. Although it is possible that the verdicts might at times end up with suboptimal justice and the so-called “lack of causation” might occasionally turn out to be an inappropriate judgment of medical appraisal, the concept is still important if negligence is confirmed by appraisal, as defendants may have the chance of winning the trial by proving lack of causation.

Finally, although 87.5% of the studied cases were acquitted, in our study, it took residents 6.22 years to achieve a final verdict. Considering the time-consuming and tortuous experience [23], physicians still lost even though they finally won the trial. Therefore, the best policy regarding malpractice claims is to avoid them.

Through verdict analysis and learning from previous medicolegal pitfalls, we believe that the risk of litigation could be reduced. For example, residents should seek timely assistance from their supervisor and communicate with patients and pay more attention to their needs. Unfortunately, current medical curricula and training programs often lack sufficient medicolegal training for residents, and their malpractice knowledge is insufficient [4, 15, 16]. Malpractice cases should be openly discussed to achieve maximal educational benefit. Further large-scale systemic reviews of previous malpractice cases are necessary in order to design a novel curriculum that improves the medicolegal training of residents.

### 4.1. Limitations

Several limitations should be noted in our study. First, we analyzed closed criminal court verdicts, but cases withdrawn due to court settlements were not available in the database, which might cause an underestimation of the actual number of malpractice claims. Second, the clinical charts were not analyzed because plaintiffs and defendants in the verdicts database were deidentified. However, detailed information on the clinical process and the statements of defendants and plaintiffs were all documented in verdicts; we believe that this was sufficient to identify the primary dispute/error leading to lawsuit, which was the most important learning objective in our study. Third, detailed information on plaintiffs or defendants was not documented in verdicts; thus, further analysis of the risk factors of lawsuits was not possible. Finally, closed medical claim analysis represents only the first step in patient safety, and further intervention studies based on information derived from court verdicts are warranted to prevent litigation.

## 5. Conclusion

Although residents are still doctors in training, they were still involved in criminal litigation cases and were found guilty in 12.5% of verdicts in our study. Supervising attending physicians were codefendants in 82.5% of these cases. Criminal medical disputes were mostly related to performance errors and took 6.22 years to conclude. Performance errors accounted for most closed criminal malpractice cases involving residents, with sepsis and operation/procedure complications accounting for 70% of such errors. To reduce litigation risk, clinical practitioners should be aware of these experiences and avoid similar medicolegal pitfalls.

## Figures and Tables

**Table 1:** Number and percentage of physicians involved in 40 malpractice claims in each specialty group^a^.

	Residents *N* = 54^b^	Guilty sentence *N* = 5	Attending physicians *N* = 51	Guilty sentence *N* = 4
Internal medicine	13 (24.1)	1 (20.0)	12 (23.5)	1 (25.0)
Surgery	21 (38.9)	1 (20.0)	18 (35.3)	0
Obstetrics	4 (7.4)	0	3 (5.9)	0
Pediatrics	7 (13.0)	0	4 (7.8)	0
Emergency medicine	7 (13.0)	2 (40.0)	9 (17.6)	1 (25.0)
Other^c^	2 (3.7)	1 (20.0)	5 (9.8)	2 (50.0)

^a^Data are presented as number of defendants (%). ^b^One radiology resident who committed suicide before the final verdict has been excluded. ^c^One neurology resident and one radiology resident were sued, and the radiologist was found guilty. One dermatology, one neurology, and two radiology attending physicians were sued, and two radiologists were found guilty.

**Table 2:** Characteristics of medical malpractice claims according to guilty or acquitted verdicts^a^.

		All claims *N* = 40	Guilty *N* = 5	Acquitted *N* = 35	Percentage of lost claims^b^
Incident-to-litigation closure (months)		77.2 ± 34.1	95.4 ± 27.9	74.6 ± 34.5	NA

Patient outcome	Death	37 (92.5)	5 (100.0)	32 (91.4)	13.5
	Grave injury	3 (7.5)	0	3 (8.6)	0

Medical institution	Medical center	25 (62.5)	2 (40.0)	23 (65.7)	8
	Regional hospital	15 (37.5)	3 (60.0)	12 (34.3)	20.0

Appraisal result	Appropriate	26 (65.0)	0	26 (74.3)	0
	Controversial	7 (17.5)	2 (40.0)	5 (14.3)	28.6
	Negligent	7 (17.5)	3 (60.0)	4 (11.4)	42.9

^a^Data are presented as patient number (%) or mean ± standard deviation. ^b^Defined as the number of loss claims divided by the number of total claims (%).

**Table 3:** Summary of negligent behaviors in defendants found not guilty.

Specialty	Major disputed behavior and acquittal reason
Not guilty due to lack of causation	
Neurology	Delay of four hours to detect abnormal troponin-I data performed in emergency department and delayed cardiology consultation in admitted stroke patient. Patient suddenly collapsed due to non-ST-elevation myocardial infarction three hours after data detection. No causation was judged by court because early consultation and diagnosis would have had minimal impact on the sudden cardiac arrest episode
General surgeon	84-year-old patient with history of diabetes mellitus and coronary artery disease suffered from incarcerated hernia. Operation was delayed 12 hours due to full operation room capacity. Patient developed myocardial infarction with cardiogenic shock one-hour after operation and expired eight hours later. Court judged the delayed operation had no causation in the myocardial infarction occurrence
Internal medicine	Diabetes mellitus patient developed spiking fever and duty resident ordered antipyretic by telephone without evaluating patient. Defendant evaluated patient 1.75 hours later, but patient developed respiratory failure and sudden cardiac arrest during the management. Defendant was found not guilty because the cardiac arrest episode was inevitable even if the evaluation had been earlier

Not guilty due to supervision	
Pediatrics	Resident prescribed high dose of valproic acid in seizure patient resulting in death caused by valproic acid overdose. The prescription was under the direct instructions of attending physician and was repeatedly confirmed by resident. The process of confirmation was well documented on the chart. Resident was acquitted while the attending physician was found guilty

**Table 4:** Numbers and percentages of primary dispute types resulting in litigation categorized by disease^a^.

	Total *N* = 40	Diagnostic error *N* = 12	Performance error*N* = 28
Guilty cases	5 (12.5)	3 (25)	2 (7.1)
Negligent behavior by appraisal	7 (17.5)	3 (25)	4 (14.3)

Diseases			
Sepsis	15 (37.5)	3 (25.0)	12 (42.9)
Operation/procedure complications	10 (25.0)	2 (16.7)	8 (28.6)
Acute coronary syndrome	3 (7.5)	2 (16.7)	1 (3.6)
Peripartum complications	3 (7.5)	0	3 (10.7)
Intracranial hemorrhage	3 (7.5)	2 (16.7)	1 (3.6)
Other^b^	6 (15.0)	3 (25.0)	3 (10.7)

^a^Data are presented as number of patients (%). ^b^Other diseases included colon perforation, delayed traumatic hemothorax, cirrhosis with gastrointestinal bleeding, valproic acid overdose, valproic-acid-induced toxic epidermal necrolysis, and malaria infection induced by inadequate sterile procedure during computed tomography.

**Table 5:** Imprisonment sentences and summary of negligent behaviors in the five guilty verdicts.

Specialty	Imprisonment sentences	Negligent behavior
Diagnostic error		
Emergency medicine	2 months	Failure to diagnose iatrogenic-colon-perforation-related peritonitis in patient receiving colonoscopy one day before emergency department visit. Resident performed fleet enema under the impression of ileus, which was thought to be a worsening factor for peritonitis by medical appraisal
Emergency medicine	5 months	Failure to reevaluate patient and repeat electrocardiogram (ECG) after patient presented with persisting chest pain and normal initial ECG and troponin-I. Patient collapsed due to ventricular fibrillation before blood test of Troponin-I follow-up
General surgery	6 months	Resident performed neck central venous catheter insertion in pancreatic cancer patient and the subsequent chest film was normal. Patient developed progressive chest pain and collapsed due to delayed massive hemothorax 3 hours later. Resident repeated patient evaluation during the process but was judged guilty because of 1) failure to identify delayed hemothorax and repeat X-ray and 2) failure to seek assistance of duty attending physician

Performance error		
General medicine	2 months	Inappropriate management of patient with severe septic shock including delayed intubation, inadequate vital signs monitoring, absence of artery blood gas exam for ventilator adjustment, and inappropriate antibiotics selection
Radiology	12 months	Resident followed disinfection protocol inadequately (failure to replace computed tomography equipment) during contrast injection resulting in cluster malaria infection. Six patients had malaria infection and four of them died. Two attending physicians were also found guilty because of failure to correct the inappropriate protocol

## Data Availability

Data are available on request due to privacy/ethical restrictions. The data that support the findings of this study are available on request from the corresponding author, Wu KH. The data are not publicly available because they contain information that could compromise the privacy of research participants.
